# Infertile human endometrial organoid apical protein secretions are dysregulated and impair trophoblast progenitor cell adhesion

**DOI:** 10.3389/fendo.2022.1067648

**Published:** 2022-12-14

**Authors:** Wei Zhou, Siena Barton, Jinwei Cui, Leilani L. Santos, Guannan Yang, Catharyn Stern, Violet Kieu, Wan Tinn Teh, Catarina Ang, Tarana Lucky, Joseph Sgroi, Louie Ye, Evdokia Dimitriadis

**Affiliations:** ^1^ Department of Obstetrics and Gynaecology, University of Melbourne, Parkville, VIC, Australia; ^2^ Gynaecology Research Centre, Royal Women’s Hospital, Parkville, VIC, Australia; ^3^ Priority Research Centre for Reproductive Science, School of Environmental and Life Sciences, University of Newcastle, Callaghan, NSW, Australia; ^4^ The Royal Women’s Hospital, Parkville, VIC, Australia; ^5^ Melbourne IVF, Melbourne, VIC, Australia; ^6^ Epworth HealthCare, Melbourne, VIC, Australia; ^7^ School of Medicine, Griffith University, Gold Coast, QLD, Australia

**Keywords:** endometrial organoids, infertile organoids, intra-organoid fluid, apical secretion, blastocyst attachment

## Abstract

**Introduction:**

Embryo implantation failure leads to infertility. As an important approach to regulate implantation, endometrial epithelial cells produce and secrete factors apically into the uterine cavity in the receptive phase to prepare the initial blastocyst adhesion and implantation. Organoids were recently developed from human endometrial epithelium with similar apical-basal polarity compared to endometrial gland making it an ideal model to study endometrial epithelial secretions.

**Methods:**

Endometrial organoids were established using endometrial biopsies from women with primary infertility and normal fertility. Fertile and infertile organoids were treated with hormones to model receptive phase of the endometrial epithelium and intra-organoid fluid (IOF) was collected to compare the apical protein secretion profile and function on trophoblast cell adhesion.

**Results:**

Our data show that infertile organoids were dysregulated in their response to estrogen and progesterone treatment. Proteomic analysis of organoid apical secretions identified 150 dysregulated proteins between fertile and infertile groups (>1.5-fold change). Trophoblast progenitor spheroids (blastocyst surrogates) treated with infertile organoid apical secretions significantly compromised their adhesion to organoid epithelial cell monolayers compared to fertile group (P < 0.0001).

**Discussion:**

This study revealed that endometrial organoid apical secretions alter trophoblast cell adhesiveness relative to fertility status of women. It paves the way to determine the molecular mechanisms by which endometrial epithelial apical released factors regulate blastocyst initial attachment and implantation.

## Introduction

Implantation failure relates to more than 50% of the lost pregnancies and is a major contributor to human infertility (1, 2). Implantation failure can be caused by abnormal development of embryos, endometrial receptivity and communication between the two (3). Among these, endometrial-related factors are related to two-thirds of implantation failures (1). The human endometrium undergoes menstrual-cycle-dependent changes to become receptive to an implanting blastocyst only at the mid-secretory (receptive) phase. At this time, blastocysts firmly adhere to the endometrial luminal epithelium initiating implantation (4). Inadequate adhesive capacity of the blastocyst or endometrial luminal epithelium leads to inadequate attachment of blastocysts resulting in failed implantation. The precise mechanisms controlling this event are still poorly defined largely due to inadequate systems in humans.

The preparation of blastocyst attachment occurs within the uterine cavity. Blastocysts can stay up to 3 days in the uterine cavity before they implant. During this time, both blastocysts and endometrial surface are bathed in an optimal environment mainly created by the endometrium (5). Endometrial glands and luminal epithelium secrete microRNAs and proteins apically into the uterine cavity, largely under the control of ovarian estrogen and progesterone. These factors regulate embryo and endometrial luminal epithelial function to prepare for the attachment of blastocysts (5, 6). Proteomic comparison of uterine fluid derived from fertile and infertile receptive phase endometrium has identified abnormal expression of implantation and fertility-related proteins in the infertile group (7). Although this sheds light on the etiology of implantation failure, a limitation of this approach is that in-depth proteomics analysis generally requires pooling of uterine fluid samples, given that very limited volumes of uterine fluid can be collected from each woman (8).

To study the effect of factors dysregulated in endometrial epithelium in women with infertility *in vitro*, we have previously cultured primary human endometrial epithelial cells (HEEC) as monolayers (9) and use them to define the roles of proteins and microRNA in endometrial receptivity and implantation *in vitro*. For example, miR-29c expression is abnormally elevated in the infertile human endometrial luminal epithelium (10). Through the use of HEECs, we are able to demonstrate that miR-29c overexpression impairs cell adhesive capacity *via* targeting Collagen type IV alpha 1 chain (COL4A1) (10). Although it is a valuable resource to assess endometrial epithelial cell adhesive capacity and explore therapeutic options *in vitro*, very few HEECs can be isolated per endometrial biopsy, cannot be passaged and survive for only one week in culture.

To overcome these limitations, organoids were recently developed from endometrial epithelium and show long-term expandability and the capability to recapitulate the histological phenotype where they were isolated from (11–14). Organoids maintain their responsiveness to hormones *in vitro* and model changes in the receptive phase of endometrium *in vivo*, after treatment with estrogen and progesterone (11, 12). The organoids maintain key features of the receptivity window, including pinopodes and pseudostratified epithelium (15). Electron microscopy confirms that organoids have their apical surface facing the center of the organoids and basolateral side facing outwards (12, 14). Such polarity is similar to endometrial glands *in vivo* and has recently been exploited to develop a protocol for large-scale collection of intra-organoid fluid (IOF) representing endometrial epithelial apical secretions (16).

Candidate biomarkers of endometrial receptivity that are expressed in a cycle-dependent manner, have been recorded with similar changes in organoids between estrogen only (modeling the proliferative phase) and estrogen and progesterone (modeling the receptive phase) treatments. Notable examples include Progestogen-associated endometrial protein (PAEP), Mucin 1 (MUC1), Progesterone receptor (PGR) and Forkhead box (FOX)O1 (11, 17–19). It has also been identified at a single-cell level that the proportion of secretory cells in organoids increases after estrogen and progesterone treatment, indicating a transition to a secretory status (18). One recent study has extended the usage of human endometrial organoids to develop a HEEC-like endometrial layer for blastoid (a blastocyst model) attachment investigation (20). Accordingly, organoid-derived endometrial epithelial cell monolayers treated with hormones and a WNT signaling inhibitor upregulate the expression of receptive genes that mark the receptive phase endometrium (20). The endometrial layers express both acetylated α-tubulin (ciliated epithelial cells) and FOXA2 (glandular epithelial cells) (20). The adhesive capacity of the organoid-derived monolayer is significantly impaired after the addition of the contraceptive progestin agent, levonorgestrel (20).

Despite these findings, the apical protein secretion of endometrial organoids and their function on initial blastocyst adhesion have not been previously studied. We developed organoids from women with primary infertility and normal fertility and compared their apical protein secretion profiles as well as functions on human trophoblast cell adhesion.

## Materials and methods

### Ethics

Written informed consent was obtained from each patient and the study was approved by the Human Research Ethics Committee at the Royal Women’s Hospital (03066B) and Melbourne IVF (74/19-MIVF).

### Endometrial tissue collection

All women (aged 26-42 years) had regular menstrual cycles (28-32 days), were not using intrauterine contraceptives for at least 3 months before surgery. Fertile women had proven parity (≥1 parous pregnancy) and infertile women had primary unexplained infertility defined as one year of failed conception with no apparent identifiable factor. Tissue samples were collected by curettage and examined by experienced gynecological pathologists to confirm the cycle stage and absence of apparent endometrial dysfunction. Each endometrial biopsy was cut into two pieces, one fixed in 10% formalin and the other one stored in ice-cold DMEM and processed for organoids culture within 1 h after collection.

### Cell lines

The endometrial adenocarcinoma cell line Ishikawa was provided by Dr M. Nishida (Tsukuba University, Tochigi, Japan). The HTR8/SVneo trophoblast cell line (CRL-3271) was purchased from the ATCC and cultured as in the manufacturer’s instructions. Human trophoblast progenitor cells (HTPCs) are a kind gift of Professor Susan Fisher and Dr Olga Genbacev (University of California, San Francisco) and cultured as previously described (21). HTPCs are developed from human embryonic stem cells have a similar gene expression profile to human day 5 trophectoderm (21).

### Human endometrial organoid establishment, maintenance and hormone treatments

Collected endometrial tissue was finely incised and digested with collagenase III (727 µg/mL, CLS-3, Worhtington; NJ, USA) and Dnase I (25 µg/mL, 11284932001; Sigma, MO, USA). Epithelial glands and luminal epithelium were collected, washed and resuspended in ice-cold Matrigel (356231, Corning; NY, USA). 25 µL drops of Matrigel suspension were plated into a 12 well plate (3 drops/well) and cultured under defined expansion medium (ExM) (11). Organoids were passaged after 7-10 days of culture and passage 2-10 were used for this study. The ExM favours epithelial cell growth and stromal cells are lost after 1-2 passages (18). To model the proliferative and receptive phase of endometrial epithelium, organoids were treated with hormones as previously described (11). Briefly, organoids were passaged and cultured under ExM for 4 days to reform the organoid structure. ExM was then supplemented with 10 nM E2. After 2 days treatment, organoids were divided into two groups: і) 10 nM E2 or ii) 10 nM E2+1µM MPA+1µM cAMP. After 4 days of treatments, IOF and EOF (the media organoids were cultured in) were collected for analysis as described below. Human endometrial organoids are comprised of both luminal and glandular epithelial cells. This has been confirmed by immunofluorescence staining of luminal epithelial marker acetylated α-tubulin and glandular epithelial marker FOXA2 (11, 18). Recent single cell analysis has further identified that after E2+MPA+cAMP treatment, 20.8% of the organoid cells are ciliated cells which indicate luminal epithelium (18).

### IOF and EOF collection

After hormone treatments, IOF and EOF were collected as previously described (16). The cultured ExM medium containing EOF was collected and centrifuged at 500 g for 5 min to remove cell debris. For IOF collection from both fertile (fertile IOF) and infertile organoids (infertile IOF), the remaining Matrigel domes were rinsed with ice-cold PBS and then incubated with Cell Recovery Solution (354253, Corning) at 4°C for 45 min. After incubation, organoids were collected and centrifuged at 270 g for 10 min, washed with ice-cold PBS and gently vortexed for 5 min to release IOF into 375 µL desired buffers based on downstream applications. The remaining organoid cells were collected and subjected to protein extraction and quantification by the bicinchoninic acid assay or RNA isolation using TRI Reagent.

### IOF incubation and spheroid adhesion assay

Spheroids were generated using HTR8/SVneo trophoblast cells or HTPCs to mimic blastocyst adhesion. Briefly, 2000 HTR8/SVneo cells or 2500 HTPCs were cultured into each well of a U shape low attachment 96-well plate for 2 days to form a spheroid of size similar to a human blastocyst. Spheroids were then collected and added to the endometrial epithelial monolayer to mimic blastocyst adhesion. To test if incubation of spheroids with IOF (E2+MPA+cAMP treated) changed their adhesive capacity, HTR8/SVneo trophoblast cells were first used to optimize the IOF volume condition for incubation. In brief, 2000 HTR8/SVneo cells were plated into each well of a U shape low attachment 96-well plate containing 150 µL culture medium as previously described (9) with the addition of 1%, 5% or 10% (v/v) of fertile IOF. The remaining organoid cells were also collected as above and protein quantified to ensure equal volumes of IOF added were generated from a similar population of organoids. After 2 days of culture, trophoblast spheroids were collected and transferred to Ishikawa monolayers for the spheroid adhesion assay (9). Culture medium was removed after 4 h incubation and each well was gently washed once with PBS to remove non-adherent spheroids. The remaining spheroids were then counted, and the percentage attachment was expressed as a percentage of the original spheroid number.

After optimization, 10% (v/v) IOF was used for the spheroid adhesion assay using HTPC-derived spheroids where the Ishikawa cells were replaced with receptive phase organoid-derived monolayers. To generate the monolayer, each well of the 96 well plate was precoated with a thin layer of Matrigel/DMEM/F12 (1:1). Organoids (after at least 4 days of culture) were broke up by pipetting up and down several hundred times before being transferred to Matrigel coated wells. Organoid cells were cultured in DMEM/F12 with 10% fetal calf serum (Invitrogen, CA, USA) and once cells outgrew and reached 100% confluency, HTPC spheroid adhesion was tested as described above. As controls for IOF treatment, HTR8/SVneo trophoblast cells or HTPCs were treated with cell culture medium (medium control) or 10% (v/v) fertile EOF and spheroid adhesion was determined and compared with IOF treated spheroids.

### microRNA transfection

Organoid-derived epithelial cells on Matrigel/DMEM/F12 were cultured in adherence to 70% confluence and transfected with miR-29c or scrambled control for 24 h using Lipofectamine RNAiMAX reagent and Opti-MEM (Thermo, MA, USA) according to the manufacturer’s instructions. Transfection medium was then replaced with fresh culture medium and cells were cultured for 48 h before downstream functional analyses.

### Immunohistochemistry and immunocytochemistry

Human endometrial tissues were fixed in 10% formalin, embedded in paraffin and sectioned at 4 μm thickness. Sections were then dewaxed, rehydrated, and antigen retrieved (microwaving in 10 mM sodium citrate for 5 min). Endogenous peroxidase activity was blocked with 3% hydrogen peroxide for 15 min at room temperature. Following washing in Tris-buffered saline (TBS) and non-immune blocking in 10% goat serum and 2% human serum in TBS, sections were incubated with primary antibodies overnight at 4°C (details summarized in **Supplemental Table 1**). An isotype control was included in every slide in which the non-immune antibody of the same IgG isotype was substituted for each primary antibody at the same concentration. Positive signaling was revealed *via* the avidin-biotin-diaminobenzidine system. Sections were counterstained with hematoxylin to indicate cell nuclei. Organoids were embedded in 5% agar and processed as described for endometrial tissues. For immunocytochemistry staining, organoid cells were fixed in 4% paraformaldehyde and permeabilized by incubation in 0.1% Triton X-100, cells were then treated as described for endometrial tissues.

### RNA isolation and RT-qPCR

Total RNA was isolated using TRI Reagent (T9424, Sigma) and treated with the TURBO DNA-free kit (AM1907, Thermo) to remove genomic DNA contamination. For gene targets detection, 300 ng total RNA was converted to cDNA using the Thermo SuperScript III First-Strand Synthesis kit. qPCR was performed using SYBR Green Master Mix (4367659, Thermo) on the Applied Biosystems ViiA7 system as follows: 95 °C for 10 min and 40 cycles of 95 °C for 15 s followed by 60 °C for 1 min. Gene expression was normalized to *18S*. miR-29c qPCR was conducted using Taqman miR reverse transcription kit and Universal Master Mix II (4366596 and 4440040, Thermo) according to the manufacturer’s instructions. miR-29c expression was normalized to *U6*. Relative expression levels of both genes and miR-29c were calculated using the comparative cycle threshold method (ΔΔCt). Primer sequences used are summarized in **Supplemental Table 2**.

### Mass-spectrometry analysis

Secreted proteins in IOF were concentrated *via* SpeedVac evaporation and quantified *via* bicinchoninic acid assay. Samples (25 µg each) were processed for proteomics analysis using single-pot, solid-phase-enhanced preparation as described previously (22). Mass-spectrometry results were analyzed using MaxQuant (Version 2.0.1.0). Peptides were matched against the UniProt Homo sapiens proteome database (updated June 20, 2021) for protein identification (23). The cleavage enzyme was set to Trypsin with a maximum of two missed cleavages and modifications selected were Oxidation (M) (variable) and Carbamidomethyl (C) (fixed). Proteins labeled as “only identified by site” and “reverse” were removed from the data set (24). The remaining list of proteins was filtered using criteria “Razor & unique peptides >1” and “Unique peptides >0” (24). Proteins were also quantified by MaxQuant’s built in Label-free quantification program and intensity-based absolute quantification was identified for each protein. Functional annotation and clustering of predicted proteins were performed using DAVID. Functional clusters that satisfied the criteria of “enrichment score >1” and “*P*<0.05” were considered enriched (25).

### IOF protein extraction and immunoblotting

IOF proteins were precipitated by addition of 5 times volume of ice-cold acetone and overnight incubation at -20°C. Protein pellets collected by centrifugation were air dried and resuspended in 5% sodium dodecyl sulfate buffer (containing 20 mM EDTA, 140 mM NaCl, 100 mM Tris pH 8.0 and proteinase inhibitor) for protein extraction. Proteins were quantified using a bicinchoninic acid protein assay kit and equal amounts of protein were resolved on 4–15% precast polyacrylamide gel and transferred to polyvinylidene difluoride membranes. Membranes were blocked with 5% skim milk in TBS for 1 h and incubated with primary antibody prepared in 5% skim milk (**Supplemental Table 1**). After washing three times with TBS-Tween 0.1% (v/v), membranes were incubated with appropriate horse radish peroxidase (HRP)-conjugated secondary antibodies. After additional washes in TBS-Tween 0.1% (v/v), labeled proteins were detected using a chemiluminescence kit. For quantification, appropriate bands were assessed by densitometry.

### Statistics

A minimum of three biological replicates were performed for all experiments (indicated by n in figure legends). For endometrial organoids, all the datapoints on the plots were representative of different organoid lines derived from different patients. Statistical analysis was carried out using PRISM 8.0 and student’s t-test or one-way ANOVA as appropriate. All data were checked for normal distribution using the Shapiro Wilk test and non-parametric tests were used for data that did not pass normal distribution test. Graphical data were presented as the mean ± SEM. *P*<0.05 was considered statistically significant.

## Results

### Establishment of human endometrial organoids from women with primary infertility and normal fertility

**Figure 1:**
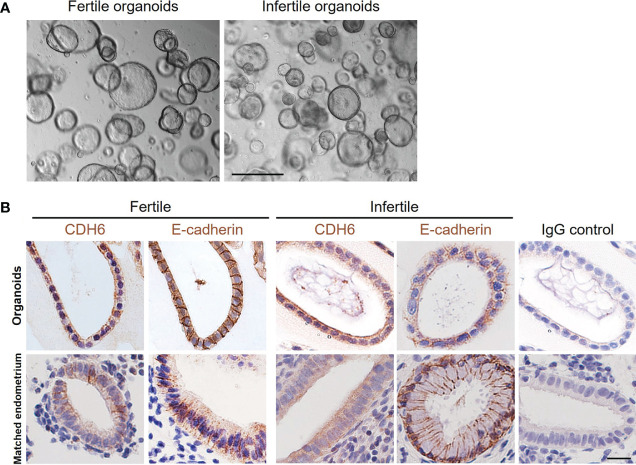
Establishment of fertile and infertile human endometrial organoids *in vitro*. **(A)** Representative images of fertile and infertile organoids under culture. The scale bar represents 200 μm. **(B)** The immunolocalization of CDH6 (tight junction marker) and E-cadherin (epithelial marker) was examined in the matched endometrial glands and organoids from the same donor. Similar localizations were recorded for all markers examined. An IgG control was included in which the non-immune antibody of the same isotype (IgG) was substituted for each primary antibody at the same concertation. The scale bar represents 20 μm.

We established organoids from both fertile and infertile endometrium. For both groups, the glandular and luminal fragments formed organoid structures within 1-2 passages with no obvious differences (**Figure 1A**). The spatial localization pattern of the tight junction protein Cadherin 6 (CDH6) and epithelial cell marker E-cadherin in organoids and matched endometrium from the same women demonstrated that both factors were similar between organoids and endometrial glands (**Figure 1B**).

### Infertile organoids show dysregulated expression of receptivity markers after hormone treatments

**Figure 2:**
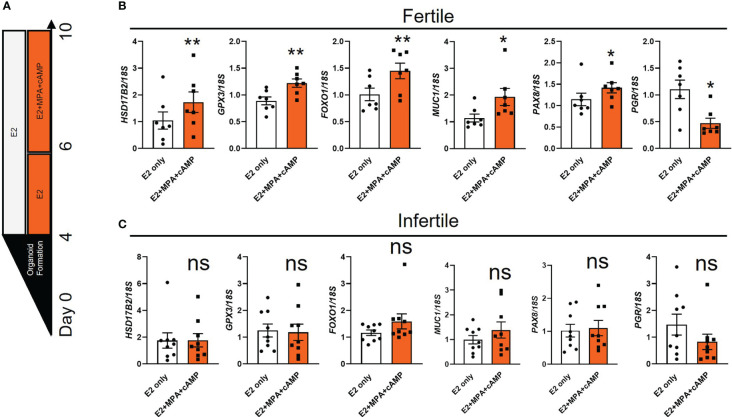
The expression of receptivity markers is dysregulated in infertile organoids after hormone treatments. **(A)** Schematic of hormone treatments for organoids. Passaged fertile and infertile organoids were cultured for 4 days in ExM to reform organoid structure before being subjected to E2 (to mimic proliferative phase) or E2+MPA+cAMP treatment (to mimic receptive phase). **(B, C)** After hormone treatments, organoid cells were collected for RT-qPCR analysis to determine the expression of receptivity markers. Expression levels were normalized to *18S* (n=7-9 biological replicates). All data were presented as mean ± SEM. *P<0.05, **P<0.01, ns, no significant difference.

Treatment of fertile organoids with estrogen or estrogen and progesterone model changes in the proliferative and receptive phases of the endometrial epithelium accordingly (11, 12, 18). To determine whether infertile organoids exhibit defects in response to hormone treatments, we examined the expression of select receptivity markers that show difference between estradiol (E2) only or E2+medroxyprogesterone acetate (MPA, a stable progestogen) +cAMP (**Figure 2A**) (11, 12, 18). Among six receptivity markers examined in fertile organoids, the expression of Hydroxysteroid 17-Beta dehydrogenase 2 (*HSD17B2*), Glutathione peroxidase 3 (*GPX3*), *FOXO1*, *MUC1* and Paired box 8 (*PAX8*) was significantly increased while *PGR* was significantly reduced (*P*<0.05) after E2+MPA+cAMP treatment, compared to E2 only (**Figure 2B**). By contrast no significant differences were identified for the expression of all genes investigated in infertile organoids between E2 only and E2+MPA+cAMP treatments (**Figure 2C**).

### Fertile organoid-derived endometrial epithelial cell monolayers respond similarly compared to primary HEEC monolayers

**Figure 3:**
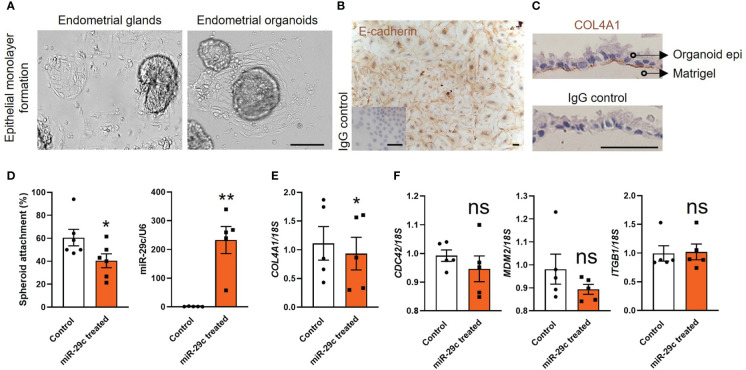
Fertile endometrial organoid sourced epithelial cells and HEECs respond similarly to miR-29c overexpression. **(A)** Endometrial epithelial cells outgrew similarly from endometrial glands and organoids. The scale bar represents 100 μm. **(B, C)** Immunocytochemistry staining of organoid derived epithelial monolayer with E-cadherin (epithelial marker) and COL4A1 (basal membrane marker). An IgG control was included in which the non-immune antibody of the same isotype (IgG) was substituted for each primary antibody at the same concertation. The scale bar represents 50 μm. **(D)** Overexpression of miR-29c in fertile organoid-derived epithelial monolayers significantly reduced HTR8/SVneo trophoblast spheroid adhesion compared to control. Similar effects on adhesion have been identified using HEECs^11^. **(E, F)** After transfection, organoid sourced epithelial monolayers were also subjected to RT-qPCR analysis to determine the expression of potential miR-29c targets as previously examined in HEECs^11^. Consistently, only *COL4A1* expression was significantly decreased after miR-29c overexpression compared to control. miR-29c expression was normalized to U6. Gene expression levels were normalized to *18S*. All data were presented as mean ± SEM (n=5-6 biological replicates). *P<0.05, **P<0.01, ns, no significant difference.

We generated endometrial epithelial cell monolayers using fertile organoids to investigate cell adhesive capacity *in vitro*. To validate our model we compared the effect of miR-29c overexpression on cell adhesion with our previous work using primary HEEC monolayers (10). Light microscopy first revealed that the endometrial epithelial cells outgrew similarly from endometrial glands and organoids (**Figure 3A**) to form a confluent cell monolayer using the same culture medium. Immunocytochemistry staining of organoid-derived epithelial cell cultures identified that E-cadherin was localized to the cell membrane and cytoplasm (**Figure 3B**). Immunocytochemistry staining of the basal membrane marker COL4A1 confirmed that the endometrial epithelial monolayers derived from the organoids stained basally (**Figure 3C**).

Functionally we identified that miR-29c overexpression in organoid-derived monolayers significantly compromised their adhesive capacity to HTR8/SVneo spheroids (blastocyst surrogates) compared to control (**Figure 3D**). Among four potential miR-29c targets examined in our previous study using primary HEECs, only *COL4A1* expression is reduced after miR-29c overexpression (10). Similarly, in organoid-derived monolayers, miR-29c overexpression significantly reduced (*P*<0.05) only *COL4A1* expression (**Figure 3E**). No significant differences were identified for the expression of Cell division cycle 42 (*CDC42*), Murine double-minute 2 homolog (*MDM2*) and Integrin subunit beta 1 (*ITGB1*) compared to control (**Figure 3F**).

### Organoid apical secretions alter trophoblast spheroid adhesion to organoid-derived monolayers *in vitro*

**Figure 4:**
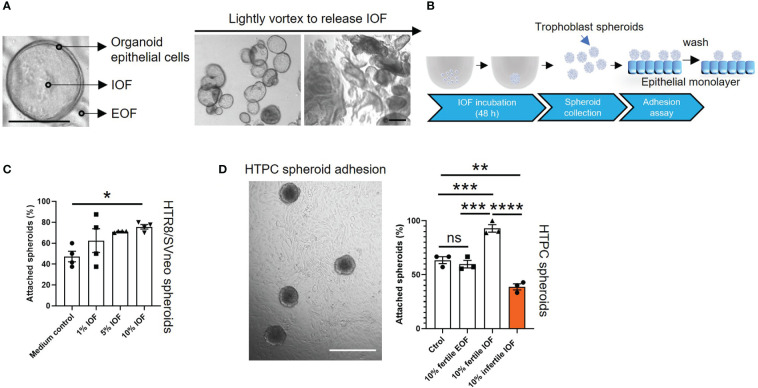
Incubation of IOF significantly changes the adhesive capacity of both HTPC and HTR8/SVneo spheroids. Fertile and infertile organoids were treated with E2+MPA+cAMP and IOF **(A)** which represent organoid apical secretions were collected to treat trophoblast cells before spheroid adhesion assay. **(A)** Representative images are presented to demonstrate that after being lightly vortexed, organoids were mildly disrupted to release IOF. The scale bar represents 100 μm. **(B)** Schematic of IOF treatment before spheroid adhesion assay. **(C)** HTR8/SVneo cells were treated with three different percentages of IOF 1%, 5% and 10% (v/v) and adhesion on Ishikawa cell monolayers tested. Only incubation with 10% (v/v) fertile IOF significantly improved HTR8/SVneo spheroid adhesion on Ishikawa monolayer compared to medium control. **(D)** After optimization, 10% (v/v) fertile or infertile IOF were used to treat HTPC spheroids and their adhesive capacity was determined on fertile organoid derived epithelial monolayer from receptive endometrium. Incubation of fertile or infertile IOF significantly changed the adhesive capacity of HTPC spheroids compared to medium control and 10% (v/v) fertile EOF control. A representative image of HTPC spheroids attaching to fertile organoid derived epithelial monolayer was shown, scale bar: 400 μm. All data were presented as mean ± SEM (n=3-4 biological replicates). *P<0.05, **P<0.01, ***P<0.001, ****P<0.0001, ns, no significant difference. IOF, Intra-organoid fluid; EOF, Extra-organoid fluid.

Endometrial glands and organoids exhibited similar apical-basal polarity (12, 14). To identify the effect of organoid apical secretions on blastocyst adhesion, organoids from both fertile and infertile groups were treated with E2+MPA+cAMP to model the receptive phase and IOF was collected using a recently optimized methodology (16). We confirmed that after lightly vortexing organoids were mildly disrupted to release IOF (**Figure 4A**). To optimize the concentration of IOF to investigate its effect on endometrial epithelial cell adhesive capacity, fertile IOF at 1%, 5% and 10% (v/v) was prepared and used to treat HTR8/SVneo trophoblast spheroids and their adhesive capacity on Ishikawa monolayers measured (**Figure 4B**). Only treatment with 10% (v/v) IOF significantly increased HTR8/SVneo spheroid adhesive capacity (*P*<0.05) to Ishikawa monolayers compared to medium control (**Figure 4C**).

10% fertile or infertile IOF was therefore used in the subsequent experiments. HTR8/SVneo spheroids and Ishikawa cells were replaced by HTPC spheroids and fertile organoid-derived monolayer, respectively. Incubation of HTPC spheroids with 10% (v/v) fertile IOF significantly increased their adhesion to fertile organoid-derived monolayers compared to medium control and 10% extra-organoid fluid (EOF, representing organoid basal secretions) control (*P*<0.001, **Figure 4D**) respectively. By contrast incubation of HTPC spheroids with 10% (v/v) infertile IOF significantly reduced their adhesion to fertile organoid-derived monolayer compared to 10% (v/v) fertile IOF (*P*<0.0001), medium control (*P*<0.01) and 10% (v/v) fertile EOF control (*P*<0.01) respectively (**Figure 4D**). No significant difference in adhesion was identified between medium control and 10% (v/v) fertile EOF control treatment groups (**Figure 4D**).

### Proteomic quantification of fertile and infertile IOF identifies differential apical secretion

**Figure 5:**
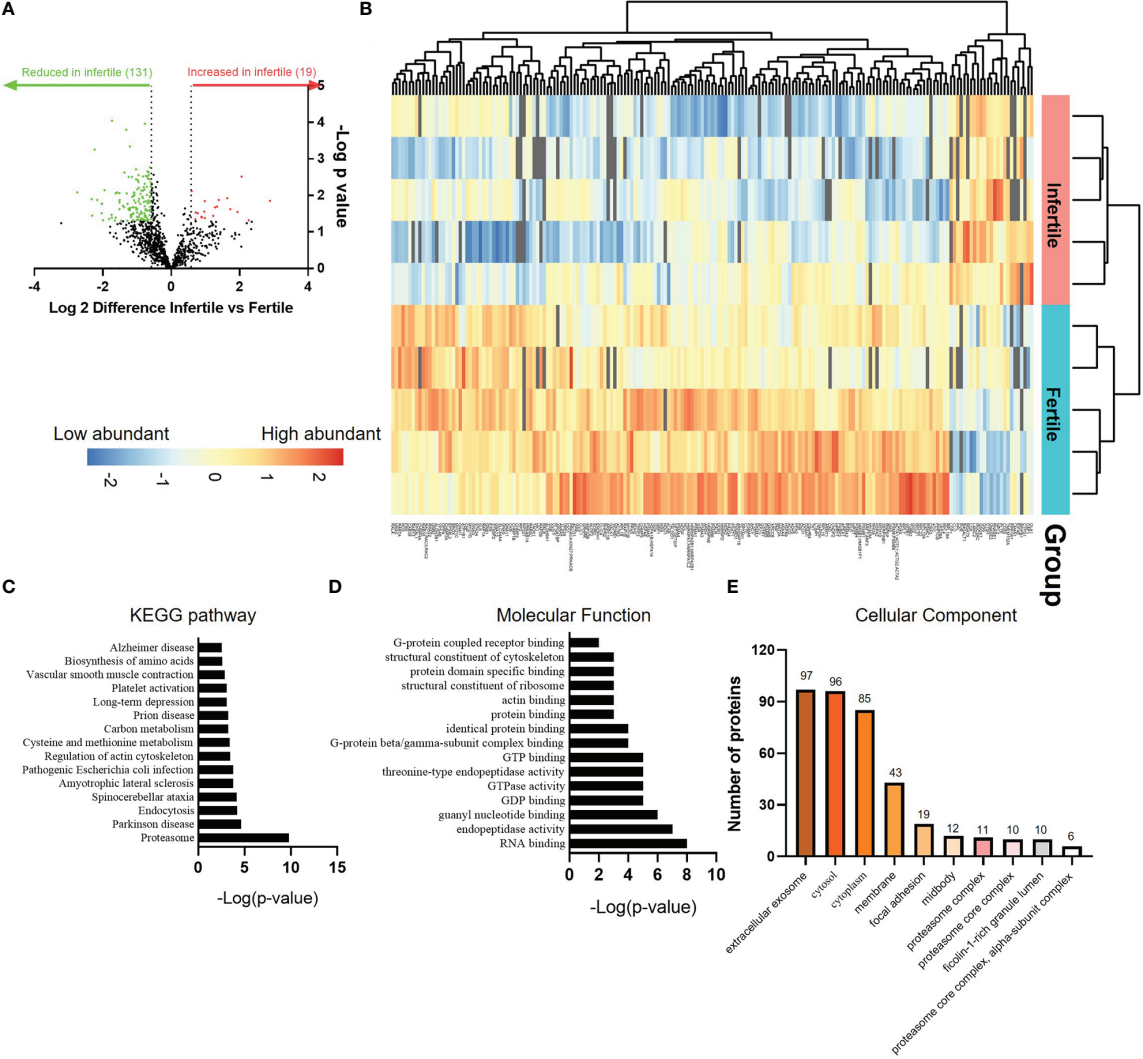
Proteomic comparison of fertile and infertile IOF after E2+MPA+cAMP treatment. **(A)** Volcano plot depicting fold changes associated with differentially expressed proteins between fertile and infertile IOF. Thresholds of ±≥1.5-fold change (*P* < 0.05) were implemented. **(B)** Heat map of the significantly differentially expressed proteins between fertile and infertile IOF with at least 1.5-fold difference. Top 15 **(C)** KEGG pathways, **(D)** GO molecular functions and **(E)** cellular components (ranked by -Log[p-value]) assigned to proteins that are significantly lower in infertile IOF with at least 1.5-fold difference compared to fertile (also see **Supplemental Tables 3**-**6**). (n=5 biological replicates). IOF, Intra-organoid fluid.

In view of the ability of IOF to alter the adhesive capacity of trophoblast spheroids, we next sought to investigate proteomic profile of fertile and infertile IOF. Organoids from both groups were treated with E2+MPA+cAMP and IOF collected for proteomic analysis. The analysis identified a total of 1150 proteins in fertile and infertile IOF. Using a threshold of 1.5-fold change and a false discovery rate of <0.05, we identified 131 proteins were decreased and 19 proteins increased in infertile IOF, compared to fertile IOF (**Figures 5A, B**; **Supplemental Tables 3** and **4**). 131 proteins that were reduced in the infertile IOF were further interrogated for enriched KEGG pathway and Gene Ontology (GO) (biological process, cellular component and molecular function) using DAVID Bioinformatics Resources (version 2021). Analysis of the top 15 KEGG pathways identified enrichment of pathways associated with cell membrane movement (endocytosis and regulation of actin cytoskeleton), infections (Pathogenic Escherichia coli infection) and protein metabolism (proteasome, cysteine and methionine metabolism and biosynthesis of amino acids) (**Figure 5C**; **Supplemental Table 5**). Similarly, analysis of the top 15 GO molecular function categories returned enriched terms related to cell membrane movement and protein binding (actin-binding, structural constituent of cytoskeleton, protein domain specific binding and G-protein beta/gamma-subunit complex binding) (**Figure 5D**; **Supplemental Table 6**). These enriched pathways and molecular functions have been consistently identified in day 16-21 normal human endometrial fluid samples as enriched categories (8), suggesting the physiological relevance of IOF. Common proteins between IOF and endometrial fluid were underlined in **Supplemental Table 3**. The most dominant GO cellular component category was extracellular exosome, with 74% of the decreased proteins (97/131) in infertile IOF mapped to this category (**Figure 5E**; **Supplemental Table 6**).

**Figure 6:**
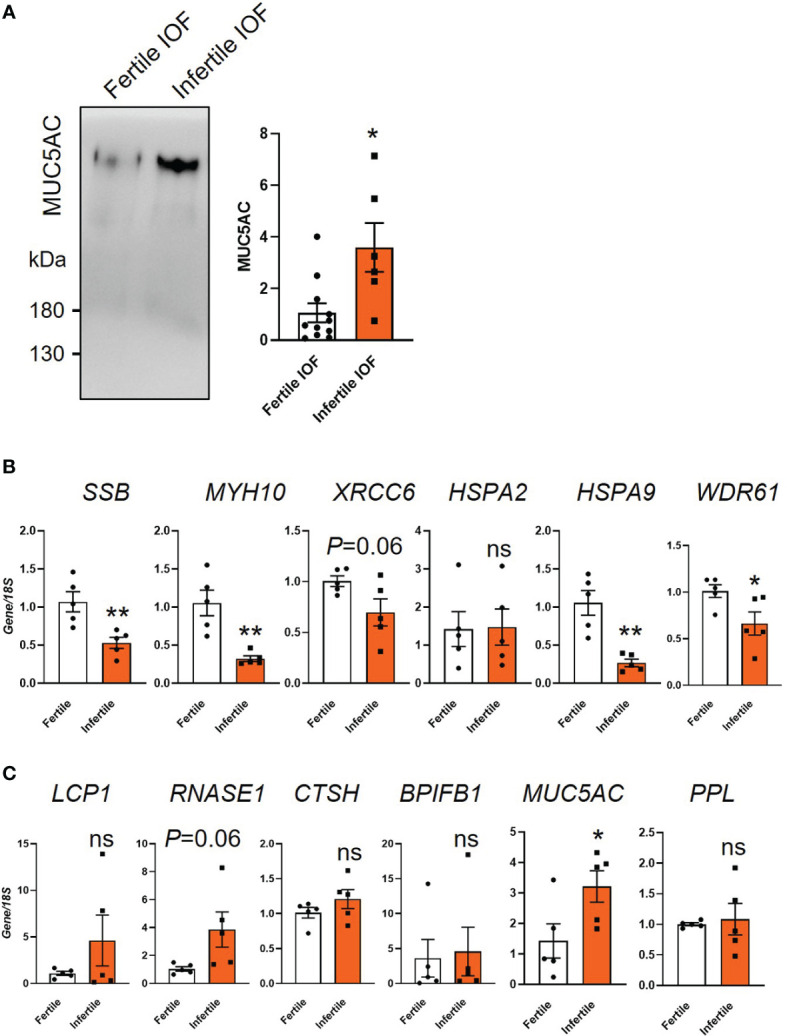
Examination of candidate proteins identified by mass spectrometry. **(A)** Immunoblot validation of MUC5AC expression in fertile and infertile IOF. Equal amounts of protein were loaded for examination (n=6-11 biological replicates). **(B, C)** qPCR examination of the production of candidate proteins in organoid cells. Top 6 significantly decreased **(B)** and increased **(C)** proteins ranked by fold change in infertile IOF compared to fertile IOF were selected for analysis. Gene expression levels were normalized to *18S* (n=5 biological replicates). All data were presented as mean ± SEM. *P<0.05, **P<0.01, ns, no significant difference; IOF, Intra-organoid fluid.

We confirmed the differential secretion of MUC5AC between fertile and infertile groups by immunoblotting. Similar to the proteomics data, MUC5AC was significantly increased in infertile IOF compared to fertile IOF (*P*<0.05, **Figure 6A**). Confirmation of the proteomics data at the mRNA level was also investigated. The organoid cells after IOF collection were subjected to qPCR for the top six proteins that were differentially produced between fertile and infertile IOF (**Supplemental Tables 3** and **4**). Among the top decreased proteins in infertile IOF, the expression of Small RNA binding exonuclease protection factor la (*SSB*), Myosin heavy chain 10 (*MYH10*), Heat shock protein family A (Hsp70) member (*HSPA*)9 and WD repeat domain 61 (*WDR61*) was significantly decreased in infertile organoid cells compared to fertile organoid cells respectively (**Figure 6B**). No significant changes were identified in the expression of X-ray repair cross complementing 6 (*XRCC6*) and *HSPA2* (**Figure 6B**). Among the top six proteins that showed a significant increase in infertile IOF, the expression of Mucin 5AC (*MUC5AC*) was significantly increased in infertile compared to fertile organoid cells (*P*<0.05), while no significant differences were identified in the expression of Lymphocyte cytosolic protein 1 (*LCP1*), *RNASE1*, Cathepsin H (*CTSH*), BPI fold containing family b member 1 (*BPIFB1*) and Periplakin (*PPL*) between groups (**Figure 6C**).

## Discussion

The results in this study demonstrate that apical secretion of endometrial organoids are differentially altered between women with infertility and normal fertility. Comparison of differential proteins in fertile and infertile IOF with uterine lavage revealed an overall similarity and enrichment in cell adhesion related functional categories. Many of the proteins were similarly differentially expressed in organoid cells at the mRNA level suggesting that they were transcriptionally regulated. Functionally, we demonstrated that incubation of apical secretions from infertile organoids after hormone treatment (to model receptivity) impaired HTPC spheroid adhesion to organoid epithelial cell monolayers. This suggests a dysregulated receptive endometrial epithelium releases factors that affect blastocyst adhesive capacity. Overall, we identified key proteins released apically by endometrial organoids that likely regulate blastocyst adhesion to the endometrial luminal epithelium. The identified factors may be useful as biomarkers of endometrial epithelial cell receptivity and embryo implantation.

To model endometrial receptivity, organoids were treated with E2+MPA+cAMP as previously published (11, 18). E2 alone treatment was used to represent the proliferative, non-receptive phase of the cycle. MPA and cAMP have been previously demonstrated to stimulate the transformation of organoids to a receptive phase phenotype that is observed in receptive phase endometrial epithelium (11, 12, 18). Our data demonstrated that six receptivity markers were dysregulated at the mRNA level after E2 and E2+MPA+cAMP treatment in infertile organoids indicating abnormal receptivity. The assessment of a wider number of genes such as those in the Endometrial Receptivity Array used to determine the optimal time for embryo transfer in an IVF treatment cycle may reveal additional dysregulated genes associated with endometrial receptivity (26). We identified variable gene expression levels between different biological replicates of infertile organoid. This suggests there is clinical heterogeneity of these infertile samples as previously reported in the endometrium of women with unexplained infertility (27). Using organoids to investigate how the endometrial epithelium prepares itself to be receptive and allows blastocysts to firmly adhere and implant is a targeted approach that until recently was difficult to achieve using primary HEECs given their short-term culture ability and variability. This targeted approach also enable us to screen proteins secreted apically by endometrial epithelium that is not possible when using whole tissue biopsies.

Organoids contain both glandular and luminal epithelial cells. A recent study developed endometrial organoid epithelial cell monolayers and investigated blastoid attachment to the monolayers (20). In the organoid-derived monolayers originating from healthy endometrium, they identified a subpopulation of cells expressed acetylated α-tubulin which marks ciliated cells in luminal epithelium and superficial glands *in vivo* (13, 20). We demonstrated that the organoid-derived monolayers exhibited polarity using the basal cell marker COL4A1 (10). We revealed similar adhesion responses between the organoid-derived monolayers and primary HEECs as we previously demonstrated (10). In this context, overexpression of miR-29c in both primary HEEC and organoid-derived monolayers significantly decreased their adhesive capacity and altered the same downstream gene targets as previously demonstrated (10). These data suggest that organoids have many similar responses to primary HEECs and are appropriate to investigate receptivity and implantation. However, it is recommended to further characterize the full epithelial polarity of organoid-derived monolayers and compare to endometrial luminal surface.

The apical-basal polarity of endometrial organoids allows the accumulation and collection of both apical and basal secretions for investigation, as demonstrated by a recent metabolomic analysis (16). Endometrial glands and luminal epithelium release their secretions apically into the uterine cavity where they can act on blastocysts and luminal surface to regulate the initial blastocyst attachment. We collected fertile and infertile IOF which represents endometrial epithelial apical secretions in the receptive phase and investigated whether there is dysregulation using an unbiased proteomics approach. Comparison of our IOF protein profiles with a recent proteomic analysis on day 16-21 normal human endometrial fluid (8) identified similar profiles. Among the 131 proteins that were downregulated in infertile IOF by at least 1.5-fold compared to fertile IOF, ~82% are identified in at least half of the endometrial fluid samples previously examined (**Supplemental Table 3**) (8). Our comparison of the top enriched Gene Ontology terms between IOF after E2+MPA+cAMP stimulation (representing the receptivity phase) and receptive phase human endometrial fluid/lavage, identified proteins in similar categories such as focal adhesion, cell-cell adhesion, cadherin binding, actin filament binding to previous studies (7, 8). Gene Ontology (cellular component) analysis revealed that 74% of the 131 decreased proteins in infertile IOF were extracellular exosome related. This is not unexpected as it is well known that proteins are released by cells *via* microparticles.

Most of the proteins whose levels significantly changed between fertile and infertile IOF by at least 1.5-fold were reduced in the infertile group (131 versus 19). Among these proteins, RCC2 regulates focal adhesion and integrin-mediated cell adhesion (28). Although the direct function of RCC2 on blastocyst adhesion has not been investigated, comparison of fertile and infertile human receptive phase uterine lavage by proteomics identifies a significantly higher level of RCC2 in the fertile group (7). Other notable examples include Dipeptidyl peptidase 4 (DPP4), a membrane-bound extracellular glycoprotein with a function to facilitate breast cancer cell adhesion in rats (29). Further evidence has suggested that DPP4 interacts with collagen and fibronectin (29, 30). The latter protein has been well characterized in the blastocysts in previous reports and shown to promote blastocyst attachment in rats (31). In humans, DPP4 has been related to glandular differentiation in the receptive phase and widely used as a receptivity marker (32, 33).

Among 19 proteins that were increased in infertile IOF, 10 of them are expressed at low to undetectable levels in healthy human endometrial epithelium, as revealed by the human protein ATLAS (both immunohistochemical staining and single-cell RNA analysis). The expression of LCP1, CTSH, BPIFB1 and Quiescin sulfhydryl oxidase 1 (QSOX1) is significantly higher in infertile receptive phase uterine lavage compared to fertile (7). Among proteins with validated functions in cell adhesion, CTSH is known as an aminopeptidase to trim the N-terminus of adhesion molecules such as Talin which then impacts its binding to integrins (34). In human prostate cancer cells, inhibition or knockdown of CTSH increases αvβ3 integrin activity and their adhesion strength (35). Mucins such as MUC1 can form an effective barrier to impact blastocyst attachment (36). We demonstrated that MUC5AC was abnormally elevated in IOF in organoids from infertile women and confirmed the finding by immunoblotting. MUC5AC is often upregulated in disease states and many of its functions may be relevant to its role in endometrial receptivity and its adhesive capacity, and implantation such as its influence on adhesion, invasion, immune cell function and inflammatory cytokines (37, 38). Low to negligible expression of MUC5AC has been observed in normal human endometrial tissue (39). However whether endometrial MUC5AC upregulation results in defective receptivity and prevents blastocyst adhesion remains to be determined.

We demonstrated that infertile organoids have dysregulated receptivity markers and treatment of HTPC spheroids with infertile IOF reduced their adhesion to the organoid-derived monolayers. Through our proteomics screen we identified dysregulation of secreted proteins in ‘receptive phase’ organoids from infertile women suggesting that at least some of these proteins may have caused the abnormal adhesion of the HTPC spheroids to the organoid-derived monolayers. Some of the identified proteins were also found to be dysregulated within the cells at the mRNA level suggesting that they may be regulated transcriptionally. It remains to be determined which of the proteins identified to be dysregulated in endometrial epithelial cell apical secretions.

In conclusion, this study has identified endometrial organoid uniquely expressed and apically secreted proteins in association with fertility status and verified the function of apical organoid secretions on HTPC spheroid adhesion. It has paved the way to determine the endometrial epithelial cell specific factors that regulate blastocyst adhesion and implantation. This is required for the development of treatment options for infertility that is currently considered unexplained.

## Data availability statement

The original contributions presented in the study are included in the article/**Supplementary Materials**. Further inquiries can be directed to the corresponding author.

## Ethics statement

The studies involving human participants were reviewed and approved by Royal Women’s Hospital (03066B) and Melbourne IVF (74/19-MIVF). The patients/participants provided their written informed consent to participate in this study.

## Author contributions

WZ conducted the experiments and drafted the manuscript. LS conducted mass-spectrometry. SB, JC and GY assisted with the mass-spectrometry data analysis. CS, VK, WT, TL, CA, JS and LY orchestrated the endometrial tissue collection. ED conceived and designed the study, contributed to data interpretation and analysis, and manuscript preparation and review. All authors contributed to the article and approved the submitted version.
