# Supplementary material for: Infertile human endometrial organoid apical protein secretions are dysregulated and impair trophoblast progenitor cell adhesion
Source: Front Endocrinol (Lausanne). 2022 Dec 14;13:1067648. doi: 10.3389/fendo.2022.1067648 (PMC9794621; doi:10.3389/fendo.2022.1067648)
Supplement: Supplementary file 1 [file Table_1.docx]

| ***Primary antibodies*** | **Final concentration / Dilution of stock solution** | | **Company** | **Catalogue N^o.^** |
| --- | --- | --- | --- | --- |
|  | Immunohistochemistry/ immunocytochemistry | Immunoblotting |  |  |
| **CDH6** | 0.5 µg/mL | N/A | Sigma | HPA007456 |
| **E-cadherin** | 0.13 µg/mL | N/A | Cell signalling | #3195 |
| **COL4A1** | 2 µg/mL | N/A | LifeSpan Biosciences | LS-C312418 |
| **MUC5AC** | N/A | 0.2 µg/mL | Thermo | MA1-21907 |

**Supplemental Table 1: Details of antibodies used throughout this study.**
