# Supplementary material for: Infertile human endometrial organoid apical protein secretions are dysregulated and impair trophoblast progenitor cell adhesion
Source: Front Endocrinol (Lausanne). 2022 Dec 14;13:1067648. doi: 10.3389/fendo.2022.1067648 (PMC9794621; doi:10.3389/fendo.2022.1067648)
Supplement: Supplementary file 2 [file Table_2.docx]

| **Supplemental Table 2. Primers used throughout this study.** | | |
| --- | --- | --- |
| **Gene** | **Forward (5'-3')** | **Reverse (5'-3')** |
| *HSD17B2* | TCTTCTCGGTGTCATGCTTCC | CAAAACTCCGGCAAATACCGT |
| *PGR* | ACCCGCCCTATCTCAACTACC | AGGACACCATAATGACAGCCT |
| *GPX3* | AGAGCCGGGGACAAGAGAA | ATTTGCCAGCATACTGCTTGA |
| *FOXO1* | TGATAACTGGAGTACATTTCGCC | CGGTCATAATGGGTGAGAGTCT |
| *MUC1* | TGCCGCCGAAAGAACTACG | TGGGGTACTCGCTCATAGGAT |
| *PAX8* | ATCCGGCCTGGAGTGATAGG | TGGCGTTTGTAGTCCCCAATC |
| *COL4A1* | AAAGGGAGATCAAGGGATAG | TCACCTTTTTCTCCAGGTAG |
| *CDC42* | GAACAAACAGAAGCCTATCAC | TTTAGGCCTTTCTGTGTAAG |
| *MDM2* | CAGCAGGAATCATCGGACTCA | ACACAGAGCCAGGCTTTCAT |
| *ITGB1* | ATTCCCTTTCCTCAGAAGTC | TTTTCTTCCATTTTCCCCTG |
| *SSB* | AATTTGCCACGGGACAAGTTT | TGTTGTTAGACGGTTCAACCTG |
| *MYH10* | TGGTTTTGAGGCAGCTAGTATCA | AGTCCTGAATAGTAGCGATCCTT |
| *XRCC6* | TTGCTTCTGCCTAGCGATACC | AAACCTGGATCATCAAACCGTT |
| *HSPA2* | AGATCGACTCGCTCTACGAGG | CGAAAGAGGTCGGCATTGAG |
| *HSPA9* | TGGTGAGCGACTTGTTGGAAT | ATTGGAGGCACGGACAATTTT |
| *WDR61* | CAGTGGAGTCTGGAGGGACAT | AGGTCCTGCATCTATGGACTT |
| *LCP1* | GGCACCCAACACTCCTATTCA | GATGACATGCCGACAATCAGG |
| *RNASE1* | ACTGTAACCAAATGATGAGGCG | GTACCTGGAGCCGTTTGTCA |
| *CTSH* | CAAGTCATGGATGTCTAAGCACC | CATTGTTGTGGGCGTTTATCTTC |
| *BPIFB1* | GGAGCTGCTAGTCAAGATCCC | CTCAGTCGTCATGTGGAACTC |
| *MUC5AC* | CAGCACAACCCCTGTTTCAAA | GCGCACAGAGGATGACAGT |
| *PPL* | GCACCAATGAGCTGTACTGG | GCTGGGGTAGTCGAGGTTG |
| *18S* | GATCCATTGGAGGGCAAGTCT | CCAAGATCCACCTACGAGCTT |
